# The Protein Kinase TPL2 Is Essential for ERK1/ERK2 Activation and Cytokine Gene Expression in Airway Epithelial Cells Exposed to Pathogen-Associated Molecular Patterns (PAMPs)

**DOI:** 10.1371/journal.pone.0059116

**Published:** 2013-03-19

**Authors:** Guy Martel, Julie Bérubé, Simon Rousseau

**Affiliations:** Meakins-Christie Laboratories, Department of Medicine, McGill University, and McGill University Health Centre Research Institute, Montreal, Canada; St. Jude Children’s Research Hospital, United States of America

## Abstract

The epithelium forms a physical barrier important to the detection of pathogens. *P. aeruginosa* infections are frequently encountered in Cystic Fibrosis lungs, lead to ERK1/ERK2 activation and contribute to tissue destruction. We report here that in bronchial airway epithelial cells (BEAS-2B), diffusible material from *P. aeruginosa* and TLR2, TLR3 and TLR5 ligands activates ERK1/ERK2 via the protein kinase TPL2 and not the growth factor receptor EGFR. Activation of TPL2 by these agonists in airway epithelial cells requires the protein kinases TAK1 and IKKβ in accordance with the previously reported model of activation of TPL2 in macrophages. Inhibition of TPL2 activity with a pharmacological inhibitor (Compound 1) not only prevented ERK1/ERK2 activation but also decreased cytokine synthesis in response to pathogen-associated molecular patterns. These results suggest that inhibition of the protein kinase TPL2 is an attractive strategy to decrease inflammation in the lungs when it is not warranted.

## Introduction

The Extracellular-signal Regulated Kinase (ERK)1/ERK2, also known as Mitogen activated Protein kinase (MAPK) 3/MAPK1, are well recognized for their roles in cell growth and differentiation occurring downstream of the v-raf-1 murine leukemia viral oncogene homolog (RAF)1 in response to growth factors [1]. However, they are also important transducers of inflammatory signals. This role is well conserved in nature, since ERK1/ERK2 confer protection to rectal infections of the nematode *C. elegans* [2]. Activation of ERK1/ERK2 in mammalian macrophages exposed to lipopolysaccharides (LPS) occurs downstream of the Tumor Progression Locus (TPL) 2 protein kinase, also known as MAPK Kinase Kinase (MAP3K) 8 [3], instead of RAF1, which is linked to growth factor activation of ERK1/ERK2. Both TPL2 and RAF1 activates ERK1/ERK2 via the phosphorylation of their direct upstream activator, MAPK kinase (MKK) 1/MKK2 (also known as MAP2K1/MAP2K2 [1]. This dichotomy in ERK1/ERK2 activation has led to great interest in pharmacological inhibition of one arm of the pathway while leaving the other unaffected.

The regulation of TPL2 activity is complex and many aspects remain unclear. TPL2 is found in unstimulated cells as a complex with TNFAIP3 interacting protein (TNIP) 2 [4] and nuclear factor of kappa light polypeptide gene enhancer in B-cells 1 (NFκB1 p105) [5]. Interaction with TNIP-2 and NFκB1 p105 stabilizes TPL2, while the interaction with NFκB1 p105 also prevents TPL2 activity towards MKK1/MKK2 [6]. Activation of TPL2 requires phosphorylation and degradation of NFκB1 p105 by the IkB Kinase (IKK) complex [7], releasing TPL2 that will autophosphorylate on Thr290 [8] and auto- or transphosphorylate on Ser400 [9]. Once activated TPL2 phosphorylates MKK1/MKK2 direct upstream activators of ERK1/ERK2. In LPS-stimulated macrophages, the cascade TPL2-MKK1/MKK2-ERK1/ERK2 is essential to the production of tumor necrosis factor (TNF)-α [3] by regulating pre-TNFα expression at the plasma membrane [10]. In hematopoietic cell lineages, TPL2 is a master regulator of ERK1/ERK2-dependent gene expression downstream of Toll-like Receptors (TLRs) [11].

Although the role of TPL2 has been well established in immune cells, its role in mediating ERK1/ERK2 activation in other cell types has been much less studied. The epithelium forms a physical barrier important in the protection and detection of pathogens. In human corneal epithelial cells infected by *P. aeruginosa*, ERK1/ERK2 activation was reported downstream of the Epidermal Growth Factor Receptor (EGFR) [12]. *P. aeruginosa* infections are frequently encountered in Cystic Fibrosis lungs and contribute to tissue destruction. TLR-stimulation and *P. aeruginosa* infection of airway epithelial cells activate ERK1/ERK2 [13], [14], but the upstream activator of this pathway is unknown. As this knowledge would be important from a biological and pharmacological standpoint, in this manuscript we have investigated which signals upstream of the ERK1/ERK2 cascade are responsible for their activation by *P. aeruginosa* in human airway epithelial cells.

## Materials and Methods

### Materials

Pam3CSK4, flagellin and Poly I:C were bought from InvivoGen (San Diego, CA, USA). 5Z-7-oxozeaenol, BI605906 and C1 were kindly provided by Professor Sir Philip Cohen (MRC PPU, University of Dundee, UK). PD184352, PD153035 and human Epidermal Growth Factor (EGF) were bought from USBiological (Swampscott, MA, USA). AG1478 was bought from Tocris Biosciences (Minneapolis, MN, USA). Pseudomonas aeruginosa diffusible material (PsaDM) was prepared and used as described previously [14].

### Antibodies

Anti-phospho Thr202/Tyr204 ERK1/ERK2 (4370) and anti-ERK1/ERK2 (9107) were used at 1/1000 dilution and purchased from Cell Signalling (Danvers, MA, USA). Anti-Raf-1 (sc-133) and anti-TPL2 (sc-720) were purchased form Santa Cruz Biotechnology (Santa Cruz, CA., USA) and used at 1/1000 and 1/500, respectively. Anti-phospho Tyr1068 EGFR (#Ab40815) was purchased from Abcam (Cambridge, MA., USA). Anti-GAPDH was purchased form Millipore (Billerica, MA. USA) and used at 1/4000 dilution. Goat anti-rabbit IgG DyLight^TM^800 (35571) and Goat anti-mouse IgG DyLight^TM^680 (35518) were used at 1/15000 dilution and were bought from Thermo Scientific (Rockford, IL, USA).

### Cell Culture

Immortalized human bronchial epithelial cells BEAS-2B cells were purchased from ATCC (Rockville, MD, USA) and cultured as described previously [14]. Cells were grown to confluence and serum starved overnight to prevent serum-dependant ERK1/ERK2 activation, prior to stimulation with agonists and/or inhibitors.

### Silencing Experiments

RAF1 siRNA (sc-29462), TPL2 siRNA (sc-35095) and siRNA transection reagent (sc-29528) were bought from Santa Cruz Biotechnology. BEAS-2B AECs were transfected in 12-wells plates according to the manufacturer’s protocols.

### Cell Lysis, RNA Extraction, Real-time PCR, Immunoblotting and ELISA

All these techniques were performed as previously described [14], [15].

### NF-κB Promoter-reporter Constructs

The NF-κB consensus response element (ggggactttcc) was synthesized in 4 copies and cloned at the XhoI/BglII sites of the pGL4.28 vector (Promega, Madison, WI, USA). The resulting vector was transformed in DH5α bacterial strain and purified with Invitrogen’s Purelink maxi prep kit (Invitrogen, Burlington, ON, Canada). pGL4.28NF-κB was transfected in BEAS-2B AECs with Fugene HD (Roche Applied Science, Basel, Switzerland) for 48 hrs and then selected for Hygromycin resistance in order to create stable cell lines. Cells were grown and stimulated as mentioned, then lysed with reporter lysis buffer (Promega). Luciferase activity in the cell extract was quantified using a Tecan M1000 microplate reader.

### Statistical Analysis

The groups were compared with the non-parametric Wilcoxon-Mann-Withney test using GraphPad Prism software (version 5.0) p-values less then 0.05 were considered to be significant. Indications of significance correspond to p-values associated to untreated cells (*) and to PsaDM-treated cells or EGF-treated cells (**#**).

## Results

### TPL2 but not EGFR or RAF1 is Required for MKK1/MKK2-ERK1/ERK2 Activation in BEAS-2B Airway Epithelial Cells Stimulated with *P. aeruginosa* Diffusible Material

**Figure 1:**
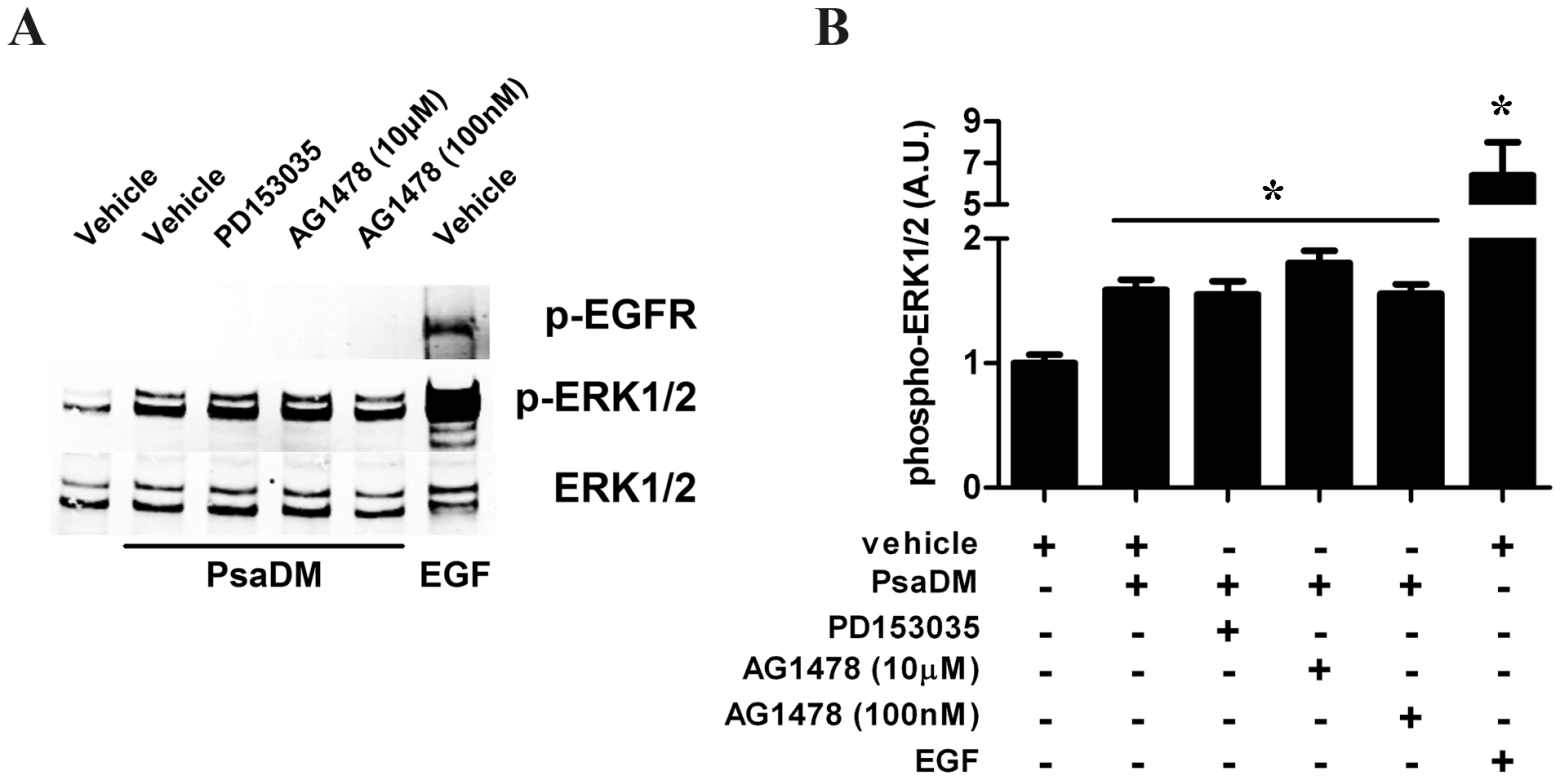
PsaDM activates ERK1/ERK2 in an EGFR-independent manner. **A–B.** BEAS-2B AECs were left untreated or exposed to 5 µg/ml PsaDM for 15 min or 50 ng/ml EGF for 30 min. Cells were lysed, and the lysates subjected to SDS-PAGE. **A.** Immunoblotting was performed with an anti-body that recognizes the EGFR phosphorylated at Tyr 1068 (p-EGFR, upper panel), an antibody that recognizes ERK1/ERK2 phosphorylated at Thr202/Tyr204 (p-ERK1/2, middle panel) or an antibody that recognizes all forms of ERK1/ERK2 (ERK1/2, lower panel). **B.** Quantitative analysis of the signal intensity obtained with an antibody recognizing only the phosphorylated forms of ERK1/ERK2 normalised to the signal obtained with antibody that recognizes all forms of ERK1/ERK2 was performed using Li-Cor infrared Odyssey imaging system.

As bacteria present in the lungs of CF patients are found mostly as intra-luminal aggregates distal from airway epithelial cells (AECs) [16], we have exposed these cells to *P. aeruginosa* diffusible material (PsaDM). Since it is not known whether PsaDM activates ERK1/ERK2 via the EGFR as reported for *P. aeruginosa* infection of corneal epithelial cells [12], we therefore prevented the activity of the EGFR using two distinct pharmacological inhibitors, AG1478 and PD153035 at 100 nM and 50 nM respectively. These concentrations were found to prevent EGFR receptor phosphorylation at Tyr1068 and ERK1/ERK2 phosphorylation at its activation motif (Thr202/Tyr204) in the immortalized bronchial airway epithelial cell line BEAS-2B stimulated with EGF (**Fig. S1A, S1B and S1C**). Blocking EGFR activity did not prevent ERK1/ERK2 phosphorylation in BEAS-2B AECs stimulated with PsaDM (**Fig. 1A and 1B**). Moreover, as in the literature AG1478 is often used at concentration 100X higher than those reported here to be required to inhibit EGFR phosphorylation, we also show that even at 10 µM, a dose likely to suppress the activity of protein kinases other than EGFR, no reduction of in ERK1/ERK2 phosphorylation is observed in response to PsaDM (**Fig. 1A and 1B**). Accordingly, although EGF led to phosphorylation of the EGFR at Tyr1068, no phosphorylation at this residue was observed in BEAS-2B exposed to PsaDM (**Fig. 1A**).

**Figure 2:**
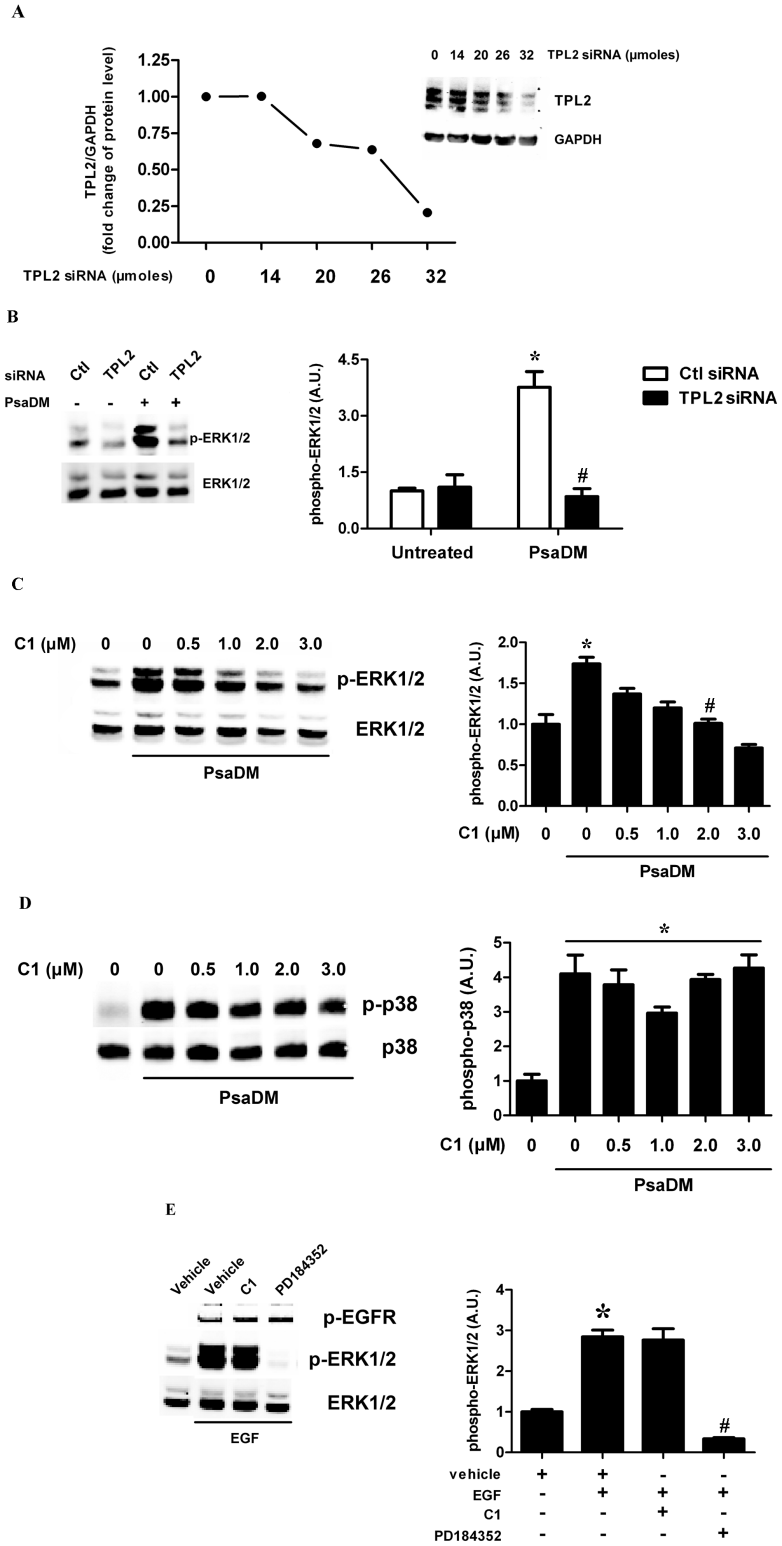
TPL2 is required for PsaDM-dependent ERK1/ERK2 MAPK phosphorylation in AECs. A. BEAS-2B AECs were transfected with increasing amounts of siRNA (0 to 32 µM) directed against TPL2 as indicated. Following lysis, 30 µg of lysates was submitted to SDS-PAGE followed by immunoblotting with an antibody that recognizes all form of TPL2. **B.** BEAS-2B AECs were transfected with control or TPL2 siRNA (32 µM) for 72 hours then left untreated or exposed to PsaDM for 15 minutes. ERK1/ERK2 was immunoblotted as in **Fig. 1**. **C–D.** BEAS-2B AECs were pre-treated 1 hour with increasing doses of the TPL2 inhibitor C1 (0–3 µM) and exposed to 5 µg/ml PsaDM for 15 minutes. ERK1/ERK2 phosphorylation was determined as in **Fig. 1** (**C**), while p38 phosphorylation was evaluated by immunoblotting with antibodies recognizing only the phosphorylated forms of p38α or antibodies that recognize all forms of p38α (**D**). **E.** BEAS-2B AECs were left untreated or pre-treated for 1 hour with vehicle, 2 µM C1 or 2 µM PD184352 (a MKK1/MKK2 inhibitor) and stimulated with 50 ng/mL EGF for 30 minutes. EGFR and ERK1/ERK2 were immunoblotted as in **Fig. 1**. Representative blots from four distinct experiments are shown (left panels). Quantitative analysis of the signals was performed and expressed as graphs (right panels).

As the MAPK pathway MKK1/MKK2-ERK1/ERK2 is not only activated by RAF1, but also by the protein kinase TPL2, we asked whether this protein kinase contributed to the activation of ERK1/ERK2 in response to PsaDM. In order to determine the contribution of TPL2 to the activation of the ERK1/ERK2, siRNA against the mRNA of this kinase was purchased and transfected in BEAS-2B AECs. By using this siRNA, we were able to knock down TPL2 protein expression in a dose-dependent manner, up to ∼80% at 32 µM (**Fig. 2A**). When BEAS-2B AECs were stimulated with PsaDM for 15 min, ERK1/ERK2 activation was suppressed in cells transfected with the TPL2 siRNA but not the non-targeted siRNA transfected cells (**Fig. 2B**). Accordingly, siRNA against RAF1 did not prevent ERK1/ERK2 phosphorylation in BEAS-2B AECs exposed to PsaDM (**Fig. S2A and S2B**). To confirm the data obtained with the siRNA, we used the pharmacological inhibitor C1 shown to block TPL2 (MAP3K8) activity [17]. In BEAS-2B AECs stimulated with PsaDM, C1 decreased the phosphorylation of ERK1/ERK2 in a dose-dependent manner, with an IC_50_ of 1 µM (**Fig. 2C**), whereas p38α (also known as MAPK14) phosphorylation was only marginally affected (**Fig. 2D**). In contrast, EGF-driven ERK1/ERK2 phosphorylation in BEAS-2B AECs was unaffected by inhibition of TPL2 with C1 (**Fig. 2E**). These results show that there exist two parallel pathways that can lead to the activation of the ERK1/ERK2 in airway epithelial cells in response to distinct agonists.

### The Protein Kinases TAK1 and IKKβ are Required for MKK1/MKK2-ERK1/ERK2 Activation in BEAS-2B AECs Stimulated with PsaDM

**Figure 3:**
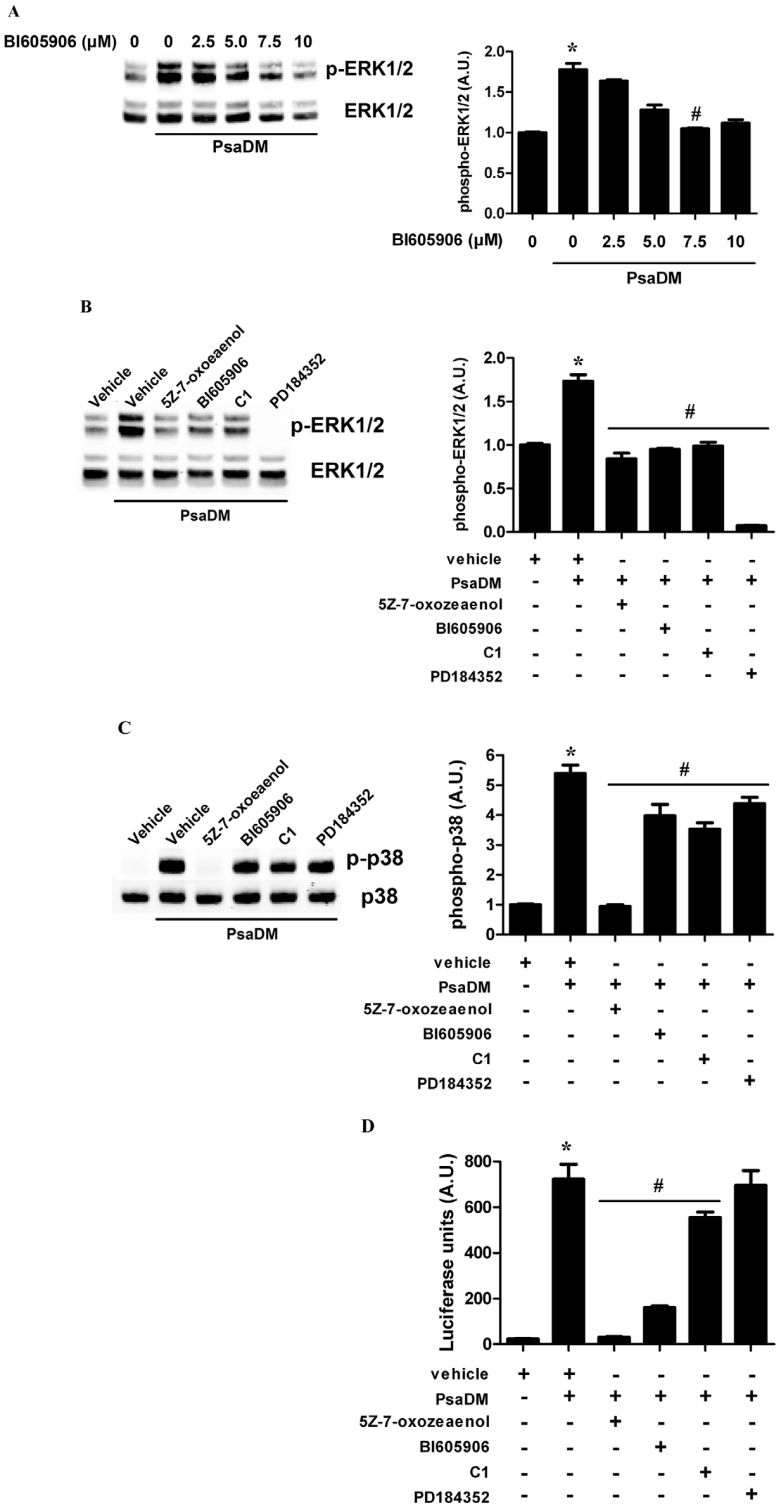
The protein kinases TAK1 and IKKβ are required for MKK1/MKK2-ERK1/ERK2 activation in BEAS-2B AECs stimulated with PsaDM. A. BEAS-2B AECs were left untreated or pre-treated 1 hour with increasing doses of the IKKβ inhibitor BI605906 (0–10 µM) and exposed to 5 µg/ml PsaDM for 15 minutes. ERK1/ERK2 phosphorylation was detected as in **Fig. 1**. **B–C.** BEAS-2B AECs were pre-treated for 1 hour with vehicle, TAK1 inhibitor 5Z-7-oxozeaenol (0.25 µM), IKKβ inhibitor BI605906 (7.5 µM), C1 (2 µM) or PD184352 (2 µM) and stimulated with 5 µg/ml PsaDM for 15 minutes. Lysates were immunoblotted for ERK1/ERK2 (**B**) and p38α (**C**). **D.** BEAS-2B AECs stably transfected with pGL4.28-NF-κB were left untreated or pre-treated for 1 hour with vehicle, TAK1 inhibitor 5Z-7-oxozeaenol (0.25 µM), IKKβ inhibitor BI605906 (7.5 µM), C1 (2 µM) or PD184352 (2 µM) and stimulated with 5 µg/ml PsaDM for 2 hours. Cells extracts were subjected to luminescence analysis. Representative blots from four distinct experiments are shown (left panels). Quantitative analysis of the signals was performed and expressed as graphs (right panels).

As mentioned in the introduction, the TLR-mediated activation of TPL2 requires the protein kinase TAK1 (also known as MAP3K7) that will in turn phosphorylate and activate the IKK complex. This will lead to the phosphorylation and degradation of NFκB1 p105, releasing TPL2 to become activated. In order to determine whether the same pathway acts in AECs, we prevented TAK1 activity using 5Z-7-oxozeaenol, a resorcylic acid lactone [18] and blocked IKKβ, also known as inhibitor of kappa light polypeptide gene enhancer in B-cells, kinase beta (IKBKB) activity using BI605906 [19]. BI605906 dose-dependently decreased ERK phosphorylation in BEAS-2B stimulated with PsaDM, with an IC_50_ of 3.75 µM (**Fig. 3A**). Moreover, pre-treating BEAS-2B AECs with 5Z-7-oxozeaenol also decreased ERK1/ERK2 phosphorylation to levels comparable to those obtained in the presence of the IKKβ or TPL2 inhibitor or in the absence of stimulation (**Fig. 3B**). The residual ERK1/ERK2 phosphorylation was suppressed by the MKK1/MKK2 inhibitor PD184352 [20] (**Fig. 3B**). In comparison, p38α phosphorylation was prevented by 5Z-7-oxozeaenol but not BI605906, C1 or PD184352 (**Fig. 3C**), whereas activation of NFκB-driven gene transcription was blocked by both 5Z-7-oxozeaenol and BI605906, but not C1 or PD184352 (**Fig. 3D**). ERK1/ERK2 basal phosphorylation was greatly decreased by PD184352 and mildly affected by 5Z-7-oxozeaenol and C1 (**Fig. S3**), suggesting that basal activity is mostly driven by the classical growth factor-mediator ERK1/ERK2 MAPK activation, whereas the PsaDM-driven inflammatory response occurs via the TAK1-IKKβ-TPL2-MKK1/MKK2-ERK1/ERK2 pathway.

### TLR-mediated ERK1/ERK2 Activation Occurs via TAK1-IKKβ-TPL2-pathway in Airway Epithelial Cells

**Figure 4:**
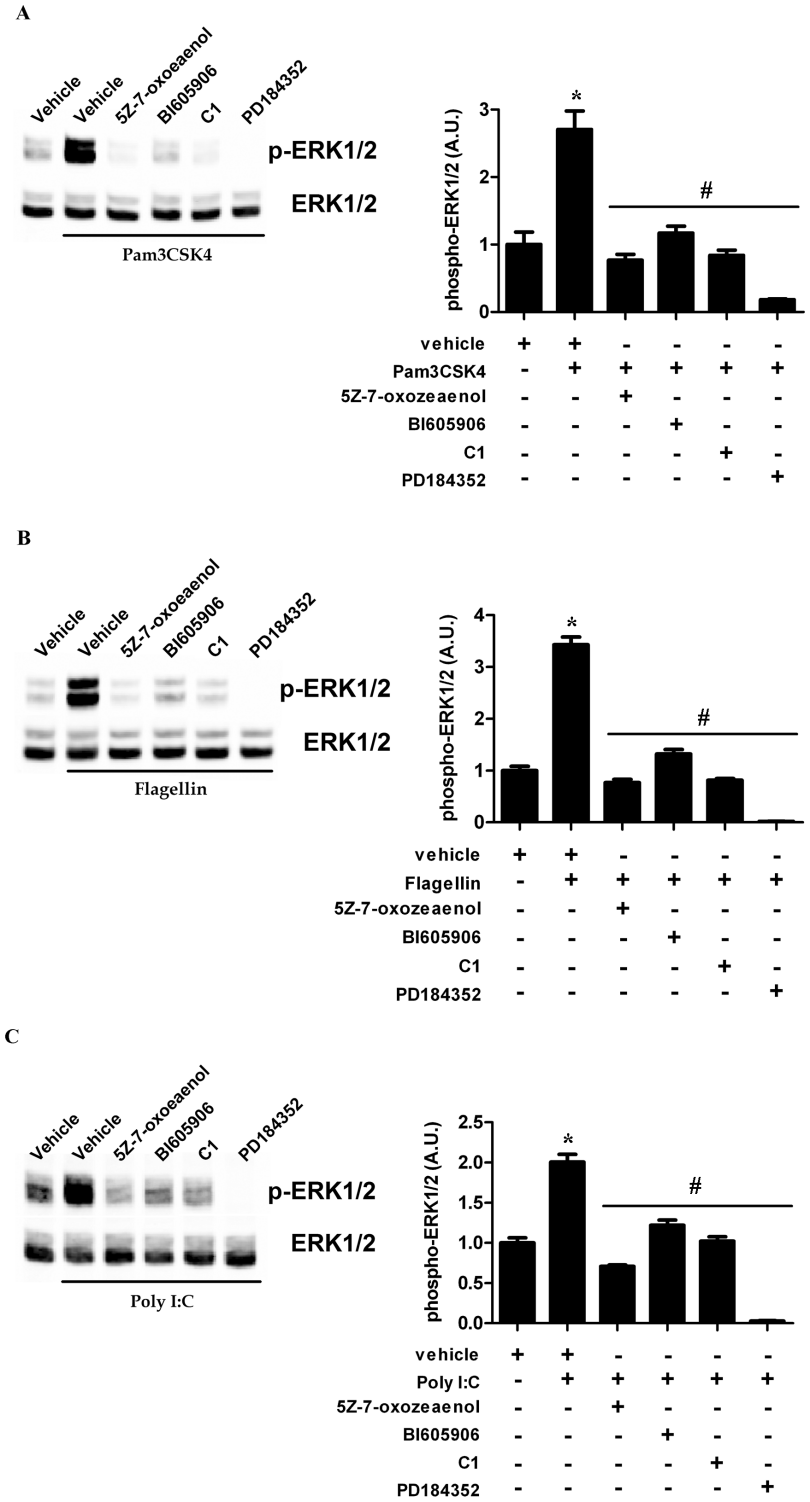
TLR-mediated ERK1/ERK2 occurs via TAK1-IKKβ-TPL2 pathway in airway epithelial cells. BEAS-2B AECs were left untreated or pre-treated for 1 hour with vehicle, TAK1 inhibitor 5Z-7-oxozeaenol (0.25 µM), IKKβ inhibitor BI605906 (7.5 µM), C1 (2 µM) or PD184352 (2 µM) and stimulated for 45 min with 1 µg/ml Pam3CSK4 (A), for 30 min with 200 ng/ml Flagellin (B) or for 75 min with 10 µg/ml Poly I:C (C). Following lysis, ERK1/ERK2 phosphorylation was determined as in **Fig. 1**. Representative blots from four distinct experiments are shown (left panels). Quantitative analysis of the signals was performed and expressed as graphs (right panels).

PsaDM activate p38α in a TLR-dependent fashion, acting mainly through TLR5 and TLR2 [15], [21], [22]. Therefore we checked whether activation of ERK1/ERK2 in BEAS-2B AECs occurs via the same mechanism when they are stimulated with isolated TLR ligands. Pam3CSK4 is a triacylated synthetic lipoprotein that binds the heterodimer TLR1/TLR2. Flagellin is a protein constituent of the flagella of motile bacteria that activates TLR5 at the cells surface. Both Pam3CSK4 and Flagellin activates downstream signaling via the MyD88 adaptor [23]. Polyinosine-polycytidylic acid (Poly I:C) is a synthetic analog of dsRNA that binds TLR3 and activates downstream signaling via the TRIF adaptor [23]. Stimulation of BEAS-2B AECs with Pam3CSK4 (**Fig. 4A**), Flagellin (**Fig. 4B**) or Poly I:C (**Fig. 4C**) led to the phosphorylation of the ERK1/ERK2 MAPK in all cases via the TAK1-IKKβ-TPL2-MKK1/MKK2 pathway (**Fig. 4**).

### Blocking TPL2 Activity Decreases Cytokine Synthesis Driven by PsaDM Stimulation of BEAS-2B AECs

**Figure 5:**
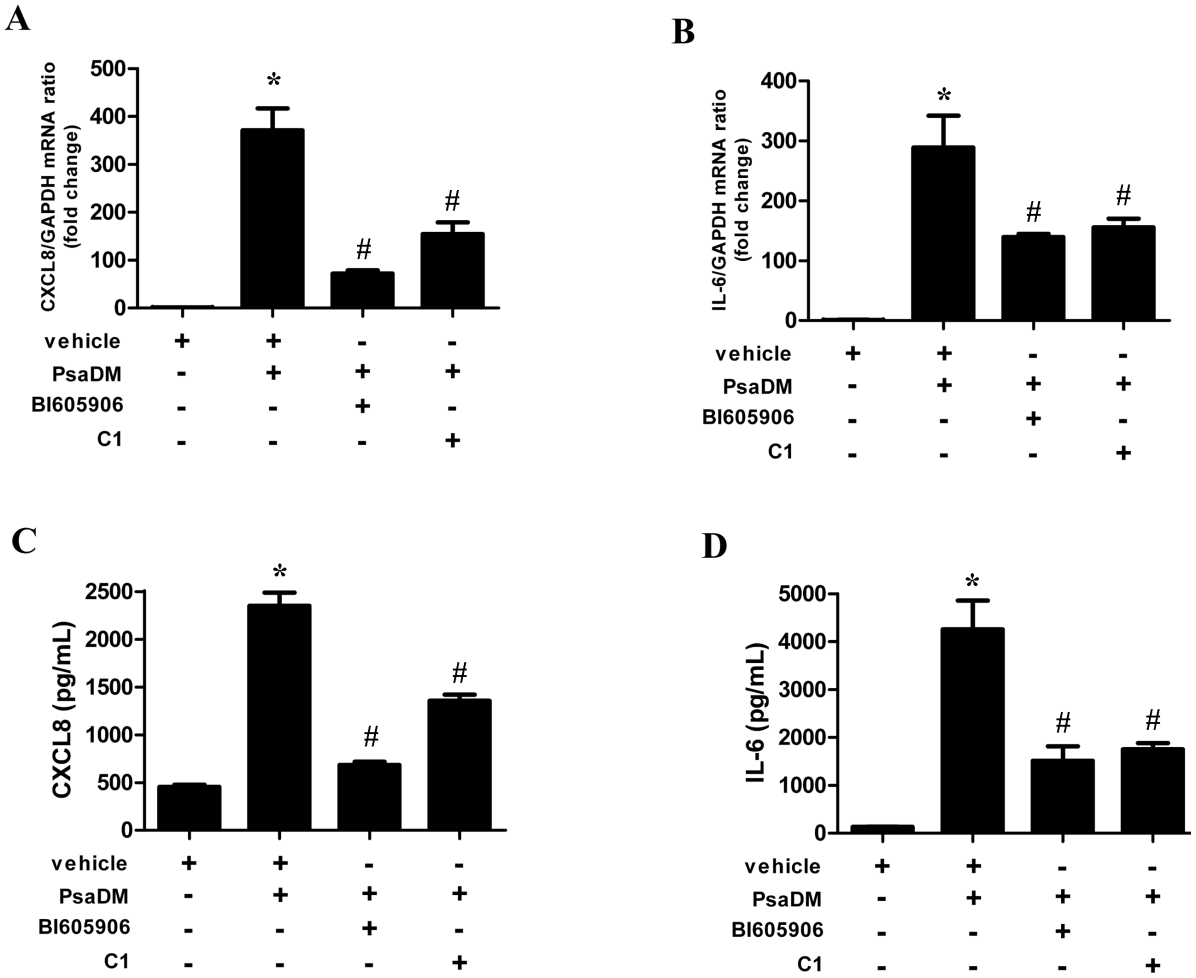
Blocking TPL2 activity decreases cytokine synthesis driven by PsaDM stimulation of BEAS-2B AECs. BEAS-2B AECs were left untreated or pre-treated for 1 hour with vehicle, 7.5 µM BI605906 or 2 µM C1 and stimulated with 5 µg/ml PsaDM for 1 hour (**A–B**) or 24 h (**C–D**). **A–B**. Total RNA was extracted and subjected to QRT-PCR for CXCL8 (**A**) and IL-6 (**B**). **C–D**. Following stimulation, the medium was collected and the amount of CXCL8 (**C**) and IL-6 (**D**) was determined by ELISA. Results from four independent experiments are shown.

A major attraction of the dichotomy of ERK1/ERK2 activation between growth factors and PAMP is the possibility of pharmacological intervention on one arm of the pathway while leaving the other intact. For example, preventing TPL2 activity could decrease inflammation in chronic inflammatory disorders. Therefore we checked whether blocking TPL2 activity with C1 in BEAS-2B AECs could decrease cytokine synthesis. In accordance with our previous reports that blocking ERK1/ERK2 activation by PD184352 decreased the mRNA levels (**Fig. S4**) and cytokine release of CXCL8 (IL-8) and IL-6 induced by PsaDM [14], [15], blocking TPL2 with C1 also decreased by 60% and 50% the transcription of both CXCL8 and IL-6 (**Fig. 5A and 5B**). Similarly, pharmacological inhibition of TPL2 impairs cytokines secretion, reducing CXCL8 and IL-6 release into the culture media by 40% and 60% respectively in response to PsaDM (**Fig. 5C and 5D**). Blocking IKKβ with the inhibitor BI605906 also decreased expression and secretion of both cytokines (**Fig. 5**).

## Discussion

**Figure 6:**
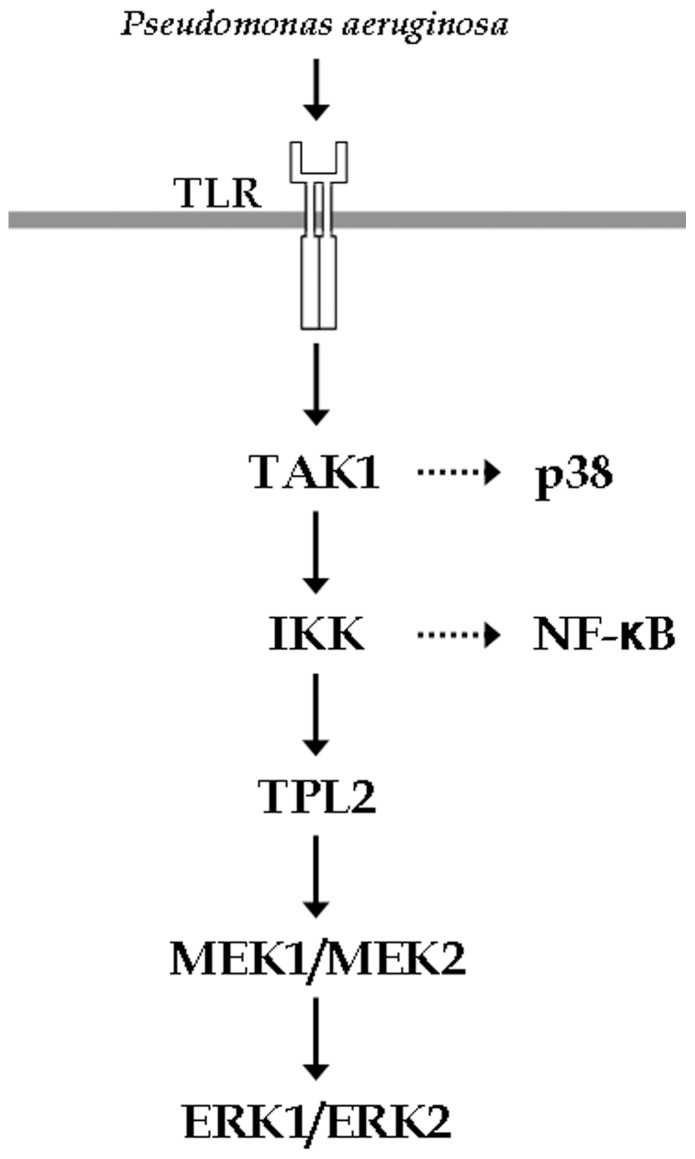
ERK1/ERK2 activation in airway epithelial cells exposed to TLR agonist occurs via the protein kinase TPL2. TLR-dependent stimulation of AECs sequentially induces the activation of TAK1, IKKβ and TPL-2, leading to the phosphorylation of ERK1/ERK2.

In this manuscript we have shown that the MKK1/MKK2-ERK1/ERK2 cascade is activated by the protein kinase TPL2 in airway epithelial cells exposed to TLR agonists and material derived from a clinical isolate of *P. aeruginosa*. In accordance with the pathway shown in macrophages [7], TPL2 is activated downstream of the protein kinases TAK1 and IKKβ (**Fig. 6**). As expected, p38α is also activated downstream of TAK1 but independently from IKKβ and TPL2, whereas NFκB activation depends on TAK1, IKKβ but not TPL2. Therefore, following TLR activation signaling diverges downstream of TAK1, with one arm that leads to p38α activation and another arm downstream of the IKK complex required for both TPL2 and NFκB activation.

In a previous report, it was shown that the MKK1/MKK2-ERK1/ERK2 MAPK cascade was activated downstream of the EGFR in corneal epithelial cells infected with *P. aeruginosa* [12]. This is in contrast with the data reported herein, where PsaDM did not lead to EGFR phosphorylation and acted via TAK1-IKKβ-TPL2. The major difference between the two models, beside the source of epithelial cells, is the live *P. aeruginosa* infection (mimicking an acute infection) versus our *P. aeruginosa* diffusible material (mimicking the chronic infection encountered in CF), which may be the reason for the differences observed.

Activation of the ERK1/ERK2 contributes to innate immunity, a function conserved in evolution [2]. Therefore, this pathway is an attractive target to decrease inflammation when it is not wanted. As the pathway regulates multiple functions, the ability to prevent or decrease its activation in response to bacterial ligands without compromising growth factor signaling is a valuable asset. We have shown that preventing ERK1/ERK2 activation using a TPL2 inhibitor, prevented ERK1/ERK2 activation in response to TLR ligands without compromising their activation in response to EGF. This supports the above notion that inflammatory and growth factor signaling can be separated in regards to ERK1/ERK2 activation. Moreover, we have shown that C1 also decreased the mRNA expression as well as the released into the media of the cytokine IL-6 and the neutrophil chemoattractant CXCL8. Therefore, these results support the idea of targeting TPL2 to decrease inflammation in chronic inflammatory lung diseases such as Cystic Fibrosis.

## Supporting Information

Figure S1 **AG1478 and PD153035 prevent ERK1/ERK2 activation by EGF**. BEAS-2B cells were pre-treated for 1 hour with increasing dose of EGFR inhibitor AG1478 or PD153035 and stimulated for 30 minutes with 50 ng/mL of EGF. Cells extracts were then subjected to EGFR and ERK1/ERK2 phosphorylation analysis. Representative blots are shown in A. Quantitative analysis of the signals was performed and expressed as graphs for EGFR phosphorylation (B) and ERK1/ERK2 phosphorylation (C).
(TIF)

Figure S2 **RNA interference of RAF1 does not prevent ERK1/ERK2 activation by PsaDM.** **A**. BEAS-2B AECs were transfected with increasing amounts of siRNA directed against RAF1 (0 to 32 µM). 75% of protein knock-down was achieves with 32 µmoles of siRNA. **B.** BEAS-2B AECs were transfected with control or RAF1 siRNA for 72 hours then left untreated or exposed to 5 µg/ml PsaDM for 15 minutes. Cells extracts were then subjected to ERK1/ERK2 phosphorylation analysis. Compared to control siRNA, targeting RAF1 had no impact on ERK1/ERK2 activation by PsaDM.
(TIF)

Figure S3 **Basal ERK1/ERK2 activation is mostly occurring independently of TAK1-IKKβ-TPL2.** BEAS-2B AECs were pre-treated for 1 hour with vehicle, TAK1 inhibitor 5Z-7-oxozeaenol (0.25 µM), IKKβ inhibitor BI605906 (7.5 µM), C1 (2 µM) or PD184352 (2 µM) and exposed to 5 µg/ml PsaDM for 15 minutes. Cells extracts were then subjected to ERK1/ERK2 phosphorylation analysis. Except for PD184352, all the others inhibitors had minor impacts on basal ERK1/ERK2 phosphorylation levels. Representative blots from four distinct experiments are shown (left panel). Quantitative analysis of the signals was performed and expressed as graphs (right panel).
(TIF)

Figure S4 **ERK1/ERK2 activation by PsaDM contributes to CXCL8 and IL-6 gene expression.** BEAS-2B AECs were left untreated or pre-treated for 1 hour with 2 µM PD184352 and stimulated with 5 µg/ml PsaDM for 1 hour. **A–B**. Total RNA was extracted and subjected to QRT-PCR for CXCL8 (**A**) and IL-6 (**B**). Results from four independent experiments are shown.
(TIF)
