# Supplementary figures and images for: The Protein Kinase TPL2 Is Essential for ERK1/ERK2 Activation and Cytokine Gene Expression in Airway Epithelial Cells Exposed to Pathogen-Associated Molecular Patterns (PAMPs)
Source: PLoS One. 2013 Mar 19;8(3):e59116. doi: 10.1371/journal.pone.0059116 (PMC3602461; doi:10.1371/journal.pone.0059116)

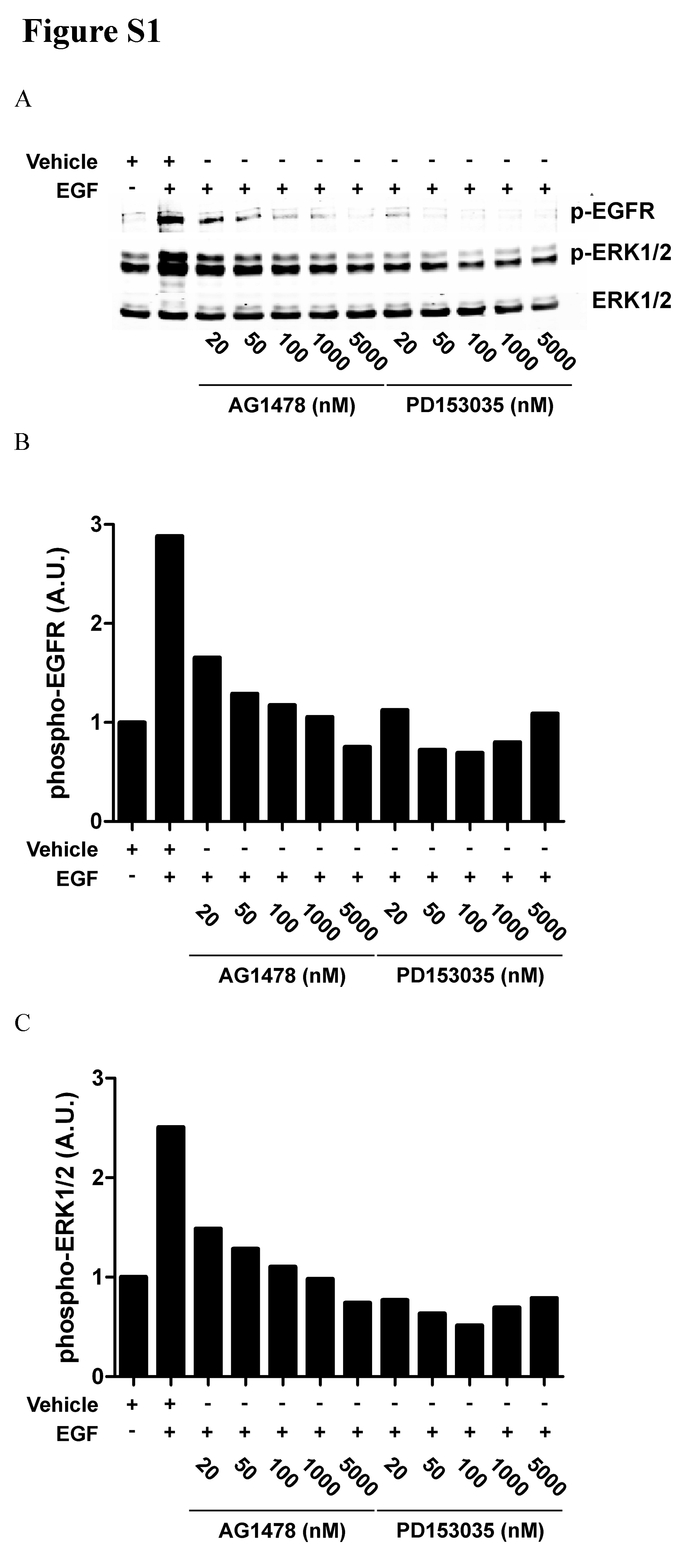

Supplement: Figure S1 — AG1478 and PD153035 prevent ERK1/ERK2 activation by EGF. BEAS-2B cells were pre-treated for 1 hour with increasing dose of EGFR inhibitor AG1478 or PD153035 and stimulated for 30 minutes with 50 ng/mL of EGF. Cells extracts were then subjected to EGFR and ERK1/ERK2 phosphorylation analysis. Representative blots are shown in A. Quantitative analysis of the signals was performed and expressed as graphs for EGFR phosphorylation (B) and ERK1/ERK2 phosphorylation (C). (TIF) [file pone.0059116.s001.tif]

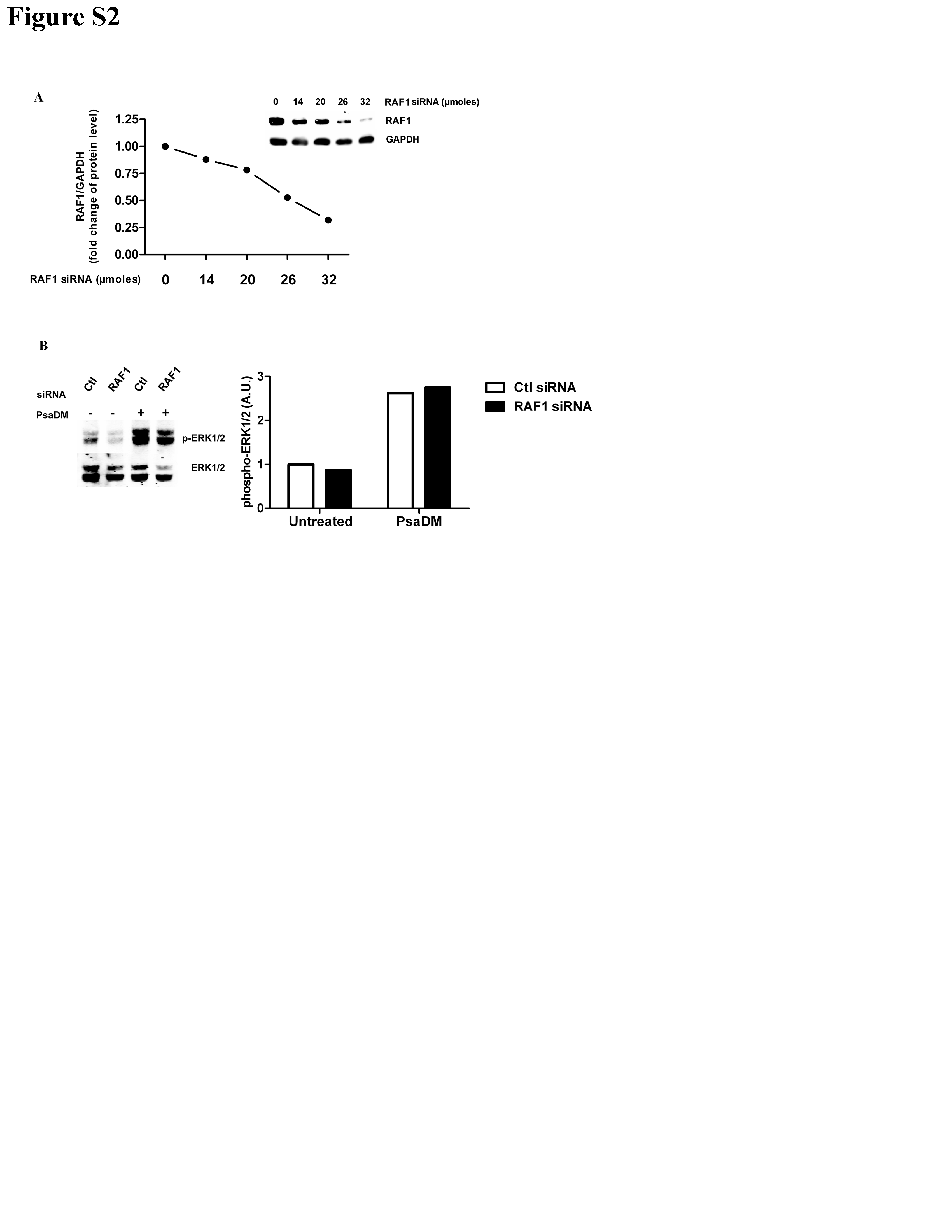

Supplement: Figure S2 — RNA interference of RAF1 does not prevent ERK1/ERK2 activation by PsaDM. A. BEAS-2B AECs were transfected with increasing amounts of siRNA directed against RAF1 (0 to 32 µM). 75% of protein knock-down was achieves with 32 µmoles of siRNA. B. BEAS-2B AECs were transfected with control or RAF1 siRNA for 72 hours then left untreated or exposed to 5 µg/ml PsaDM for 15 minutes. Cells extracts were then subjected to ERK1/ERK2 phosphorylation analysis. Compared to control siRNA, targeting RAF1 had no impact on ERK1/ERK2 activation by PsaDM. (TIF) [file pone.0059116.s002.tif]

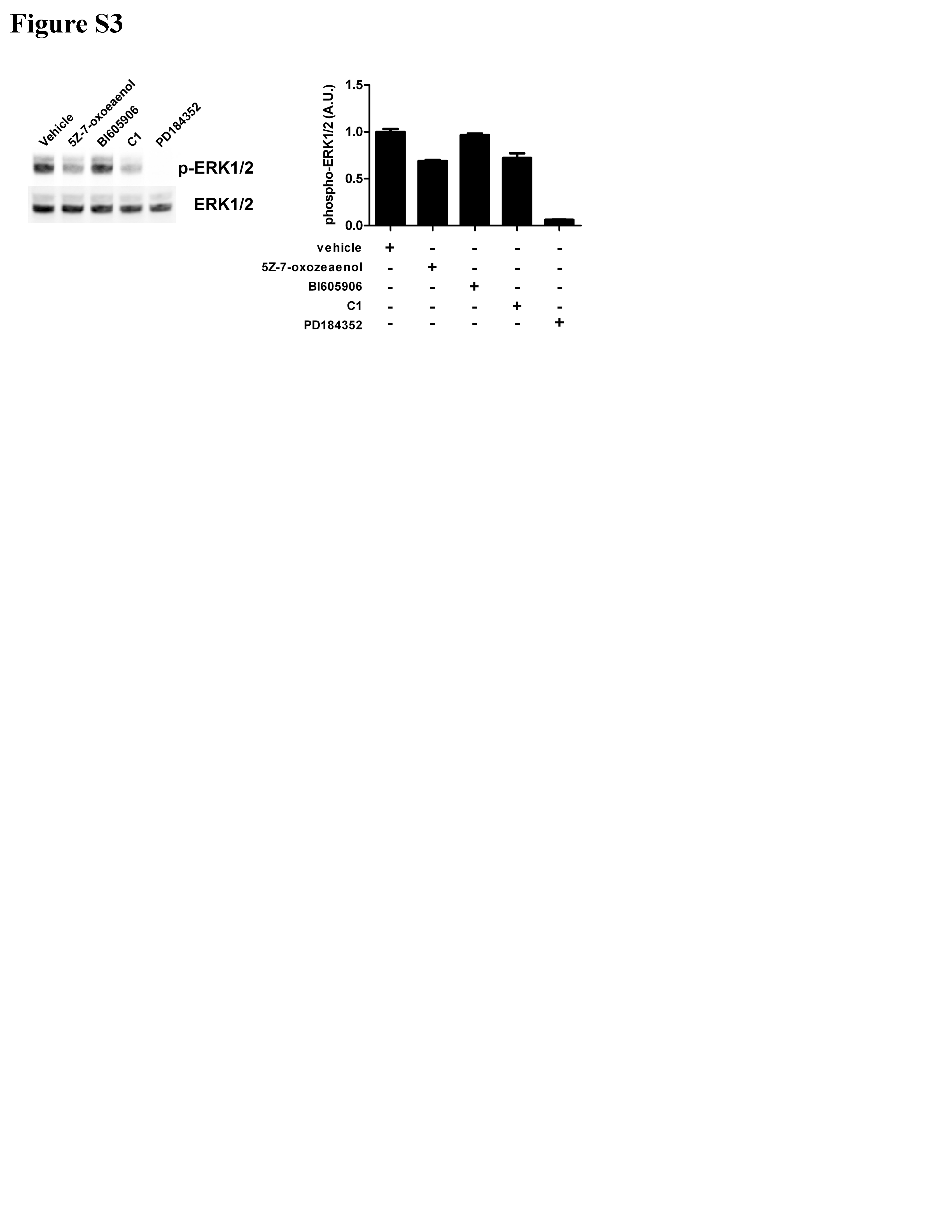

Supplement: Figure S3 — Basal ERK1/ERK2 activation is mostly occurring independently of TAK1-IKKβ-TPL2. BEAS-2B AECs were pre-treated for 1 hour with vehicle, TAK1 inhibitor 5Z-7-oxozeaenol (0.25 µM), IKKβ inhibitor BI605906 (7.5 µM), C1 (2 µM) or PD184352 (2 µM) and exposed to 5 µg/ml PsaDM for 15 minutes. Cells extracts were then subjected to ERK1/ERK2 phosphorylation analysis. Except for PD184352, all the others inhibitors had minor impacts on basal ERK1/ERK2 phosphorylation levels. Representative blots from four distinct experiments are shown (left panel). Quantitative analysis of the signals was performed and expressed as graphs (right panel). (TIF) [file pone.0059116.s003.tif]

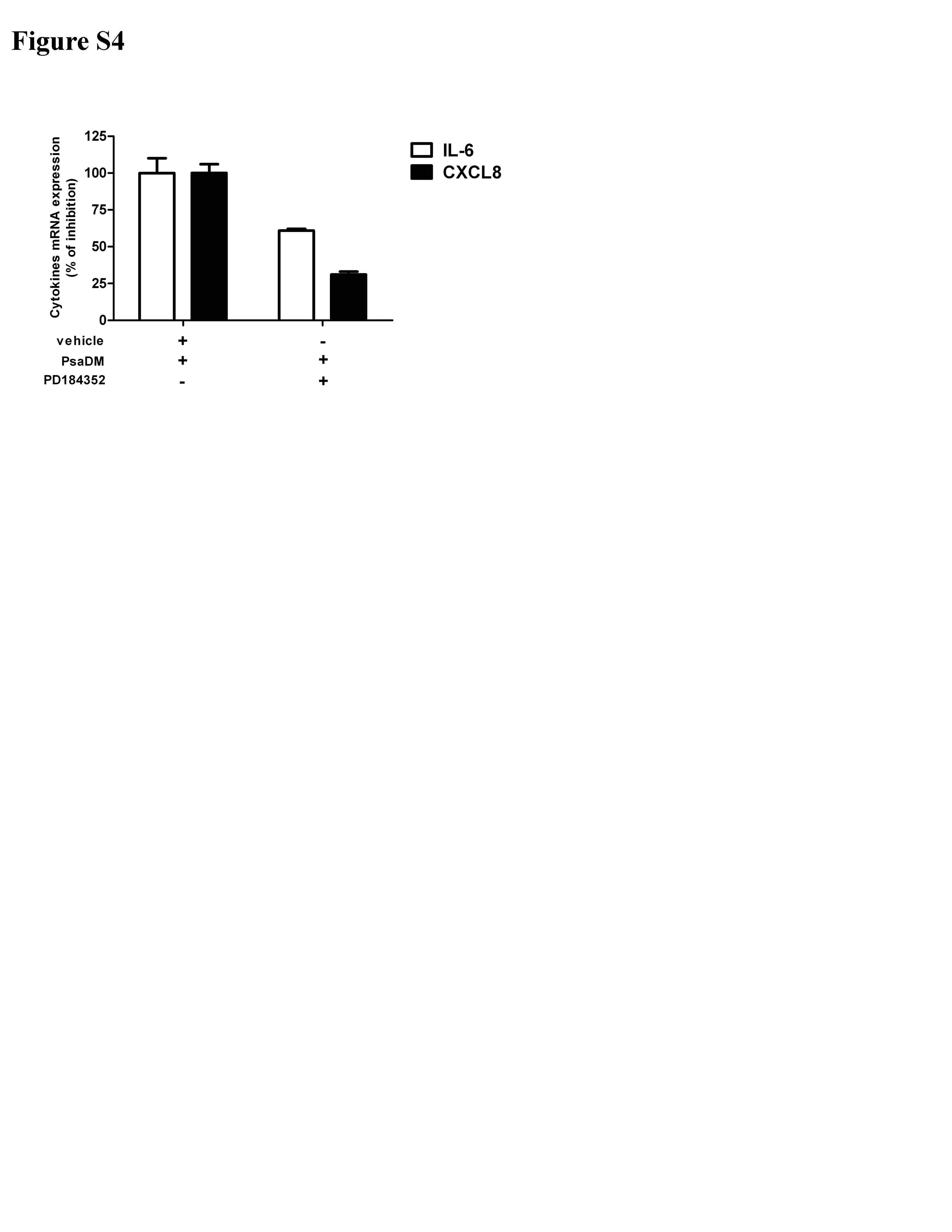

Supplement: Figure S4 — ERK1/ERK2 activation by PsaDM contributes to CXCL8 and IL-6 gene expression. BEAS-2B AECs were left untreated or pre-treated for 1 hour with 2 µM PD184352 and stimulated with 5 µg/ml PsaDM for 1 hour. A–B. Total RNA was extracted and subjected to QRT-PCR for CXCL8 (A) and IL-6 (B). Results from four independent experiments are shown. (TIF) [file pone.0059116.s004.tif]
